# Revictimisation Across Types of Interpersonal Violence: A Meta‐Regression Analysis of PTSD and Associated Factors

**DOI:** 10.1002/smi.70079

**Published:** 2025-08-06

**Authors:** Christin Kühner, Julie Emmelkamp, Anneke E. Goudriaan, Marleen M. de Waal, Kathleen Thomaes

**Affiliations:** ^1^ Department of Psychiatry Amsterdam University Medical Centers Location University of Amsterdam Amsterdam the Netherlands; ^2^ Research Department Arkin Mental Health Care Amsterdam the Netherlands; ^3^ Amsterdam Institute of Addiction Research (AIAR) Jellinek Arkin Mental Health Care Jellinek the Netherlands; ^4^ Sinai Centrum/Arkin Mental Health Care Amstelveen the Netherlands; ^5^ Department of Clinical, Neuro and Developmental Psychology Vrije Universiteit Amsterdam Amsterdam Public Health Research Institute Amsterdam the Netherlands; ^6^ Department of Youth and Family Arkin Mental Health Care Amsterdam the Netherlands; ^7^ Department of Psychiatry Amsterdam University Medical Center Location Vrije Universiteit Amsterdam the Netherlands

**Keywords:** interpersonal violence, intimate partner violence (IPV), meta‐science, methodology, post‐traumatic stress disorder (PTSD), revictimisation

## Abstract

The literature has consistently demonstrated that being the victim of interpersonal violence increases the risk of future interpersonal violence (i.e., revictimisation). In this context, post‐traumatic stress disorder (PTSD) is highly important since it has been investigated as a risk factor and consequence of revictimisation. The aim of the current study was to (a) compute a rate of revictimisation across types of interpersonal violence, and (b) examine which factors are associated with observed rates of revictimisation. We conducted a pre‐registered systematic search in PubMed, APA PsycInfo, PTSDpubs, Web of Science, and Scopus, that resulted in *N* = 1286 individual records and *n* = 19 inclusions (PROSPERO ID: CRD42023446788). Criteria for inclusion were: adult human sample, assessment of PTSD symptoms that precedes assessment of interpersonal revictimisation, published in a peer‐reviewed journal, longitudinal study design. Most studies focused on intimate partner revictimisation, followed by sexual revictimisation. The pooled rate of revictimisation was 39.2% for the overall group, and 53.4% for those experiencing intimate partner revictimisation. Only the proportion of females was positively associated with the observed rate of revictimisation. None of the other factors: presence of severe PTSD symptoms, mode of assessment (PTSD), type of sample, or duration of the follow‐up period, were associated with the rates of revictimisation. We recommend the use of gold‐standard assessment for PTSD, more representative and more clearly defined samples, and the development of a validated measure of revictimisation. For clinicians, we recommend paying attention to and providing psychoeducation about revictimisation and potential ways to prevent this. In sum, revictimisation is highly prevalent, but remains poorly understood. This gap may be addressed by rigorous methodological improvements (research) and psychoeducation (clinical practice).

## 1 Introduction

Being the victim of interpersonal violence has been linked with a plethora of negative consequences, such as post‐traumatic stress disorder (PTSD), depressive symptoms, and a higher risk for substance use (Schilling et al. 2007; Goodrum et al. 2022). Importantly, interpersonal victimization also leads to a greater risk of being the victim of subsequent violence, called *interpersonal revictimisation* (henceforth: *revictimisation*; Schilling et al. 2007; Christ et al. 2022). A meta‐analytic review has demonstrated that around 50% of survivors of sexual violence experience sexual revictimisation (Walker et al. 2019). A meta‐analysis of intimate partner violence (IPV) revictimisation also reported high rates of revictimisation in prospective studies (23.7%–50.5%; Bellot et al. 2024). Importantly, people who have been victimised are often the victims of multiple types of victimization incidents, such as physical *and* sexual abuse (Christ et al. 2022; Finkelhor et al. 2007).

A group that is especially vulnerable to a broad range of victimization *and* revictimisation incidents are people with psychiatric disorders, for example, people with major depressive disorder, and/or substance use disorder (Christ et al. 2022; de Waal et al. 2017; Maniglio 2009). Persons with a psychiatric disorder were 5.8 times more likely to experience sexual assault during the past year when compared with the general population (de Waal et al. 2017). Rates of physical assault were even 14.8 times higher in persons with a psychiatric disorder than in the general population (de Waal et al. 2017). Furthermore, PTSD is a highly important disorder in the context of revictimisation, as it has been investigated as both a risk factor for and a consequence of revictimisation in several systematic reviews/meta‐analyses (Fereidooni et al. 2023; Walker and Wamser‐Nanney 2023; Cividanes et al. 2018). PTSD is characterised by symptoms of intrusion, avoidance, negative changes in mood and cognition, and hyperarousal (American Psychiatric Association 2013). People are at increased risk of developing PTSD after experiencing interpersonal violence, underlining the importance of doing longitudinal research where the measurement of PTSD symptoms precedes the measurement of revictimisation (Goodrum et al. 2022).

However, no meta‐analysis to date has focused exclusively on the longitudinal impact of PTSD on the risk of revictimisation. Meta‐analyses so far have included both cross‐sectional and longitudinal studies, in which PTSD symptoms could have been either risk factors for or consequences of revictimisation. Moreover, as said, the meta‐analyses that have been conducted have focused on only one type of revictimisation incident (sexual/IPV; Walker et al. 2019; Bellot et al. 2024). Therefore, a knowledge gap remains regarding the predictive value of the severity of PTSD on the risk of revictimisation. Additionally, scarce attempts have been made to explain the factors that lead to differences in rates of revictimisation (see Walker et al. 2019) for an exception with a focus on sexual revictimisation). We aimed to expand the body of knowledge to different types of revictimisation incidents and the associated variance in estimates of revictimisation. First, we computed an umbrella statistic of the rate of revictimisation in studies that report on both revictimisation and PTSD symptoms. We were interested in explaining some of the variability in rates of revictimisation across studies. Specifically, we were interested in quantifying the effect of:

- the severity of PTSD symptoms in the sample, given that PTSD symptoms have been linked with revictimisation in earlier systematic reviews (Fereidooni et al. 2023; Kühner et al. Forthcoming). Based on these systematic reviews, we expected a positive association between severity of PTSD symptoms and rate of revictimisation.
- the mode of assessment of PTSD symptoms, that is, self‐report or clinician‐assessed. Prior studies have indicated that self‐report and clinician‐administered measures provide largely similar but not identical change scores in PTSD symptoms (Lee et al. 2022). Namely, self‐report measures for PTSD symptoms can lead to higher estimates than clinician‐administered measures (Geier et al. 2019), but the reverse has also been found (Monson et al. 2008; Youngstedt et al. 2022). Therefore, we examined the mode of assessment of PTSD symptoms on revictimisation rates. Due to the mixed findings in the literature, we had no prior expectations regarding the direction of this relationship.
- the type of sample under investigation, since studies on revictimisation have included a range of samples from convenience, veterans, to women staying at battered women's shelters (Najdowski and Ullman 2009; Scoglio et al. 2022; Dokkedahl et al. 2022). We expected a higher rate of revictimisation in the women staying at battered women's shelters, since prior victimization severity has been linked with the risk of revictimisation (Kuijpers et al. 2011).
- the proportion of females in the sample, given the overrepresentation of all‐female samples in the revictimisation literature (Walker et al. 2019). The only meta‐analysis examining the role of gender relied on a majority of all‐female samples and found no relationship between gender and sexual revictimisation (Walker et al. 2019). We examined a broader range of revictimisation incidents and therefore had no prior expectations regarding the direction of this relationship.
- the location/country the study was conducted in since earlier meta‐analyses have examined the effects of the location in the context of PTSD and found an effect when comparing studies conducted in Asia to those conducted in North America (Martínez and Blanch 2024). We also we knew beforehand that many studies on revictimisation were conducted in the U.S.. The U.S uses the Diagnostic and Statistical Manual for Mental Disorders Fifth Edition (DSM‐5) to diagnose/assess PTSD, whereas most of Europe uses the International Classification of Diseases and Related Health Problems (ICD‐11; American Psychiatric Association 2013; First et al. 2015). Although these diagnostic instruments greatly overlap, we wanted to examine any potential differences due to the predominant diagnostic system. We had no prior expectations regarding the direction of this relationship.

The resulting knowledge is relevant for the field of revictimisation since we examined study characteristics and their unique contributions to the observed rate of revictimisation. Therefore, researchers planning to conduct studies on revictimisation can identify study characteristics that potentially affect the rate of revictimisation observed in a study. Clinically, our findings are important since they allow us to identify groups that are particularly vulnerable to revictimisation. Consequently, knowledge of vulnerability factors can be used to design interventions aimed at preventing revictimisation.

## 2 Methods

### 2.1 Identification of Studies

We conducted a pre‐registered systematic search of the literature in accordance with the guidelines described in the PRISMA protocol (Matthew et al. 2021; PROSPERO ID: CRD42023446788). We used the following MeSH‐terms ‘Stress Disorders, Traumatic’, ‘Adult Survivors of Child Abuse’ in combination with a range of title, abstract, and author‐supplied keywords for ‘child abuse’, ‘revictimisation’, and ‘victimization’. For a full overview of the search terms per database see Supporting Information S1: Appendix 1, for the PRISMA checklist see Supporting Information S2: Appendix 2, and for the PROSPER protocol see PROSPERO. We searched for the combination of both terms in PubMed, APA PsycInfo, PTSDpubs, Web of Science, and Scopus from 1980 to September 2024. We had no access to any information that would allow us to identify individual participants. We made a pre‐selection of eight articles that we used to evaluate the effectiveness of our search strategy.

### 2.2 Inclusion of Studies

We applied the following inclusion criteria for studies resulting from our systematic search: (1) adult human sample with a mean age of 18 years or higher, (2) assessment of PTSD symptoms (either cluster‐scores or total score) according to DSM‐criteria (questionnaire or self‐report), (3) assessment of interpersonal revictimisation, that is, physical assault, sexual assault, threat of assault, emotional violence, (4) published between 1980 and 2024, (5) published in a peer‐reviewed journal, (6) longitudinal study design, (7) assessment of PTSD symptoms precedes assessment of interpersonal revictimisation, (8) everybody in the sample was victimised at the start of the study OR the authors included subgroup analyses for those who were victimised at the start of the study. We searched for studies from 1980 onward, since that was the year that PTSD was introduced as an official diagnosis in the DSM.

**FIGURE 1:**
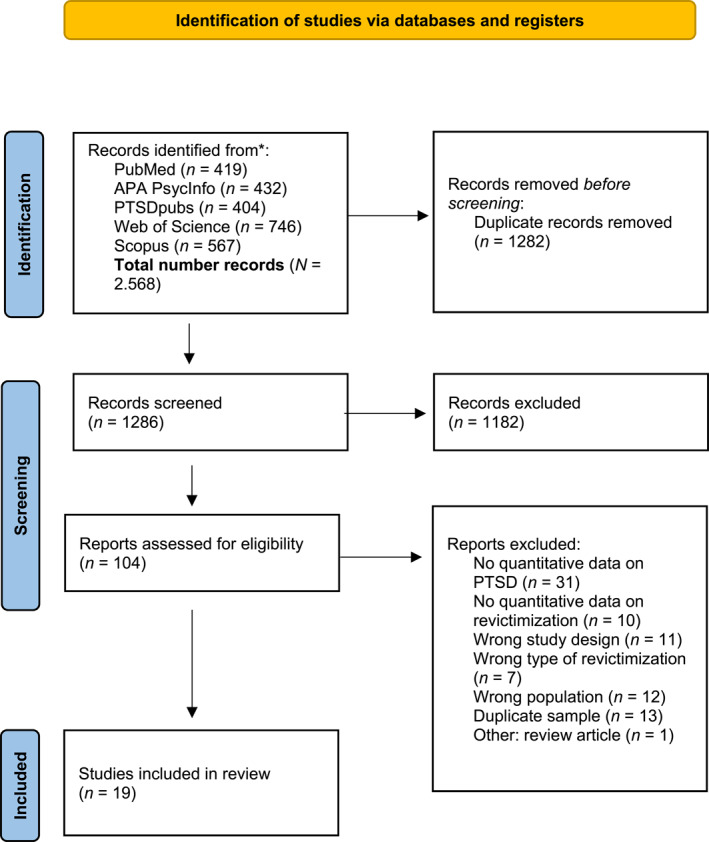
PRISMA flowchart of the systematic literature search.

We applied the following exclusion criteria: (1) no quantitative data on PTSD symptoms, (2) no rate of revictimisation reported in the article, (3) intervention study, (4) duplicate sample (we chose the article that matched the most with our research interest and provided the data we were interested in whenever two articles used the same sample), (5) fewer than 20 participants (Simmons et al. 2011). The first author (CK) screened all articles and KT (97% interrater agreement), MdW (98% interrater agreement), and AG (98% interrater agreement) each screened a third of all articles. The first author (CK) proceeded with the full‐text screening (95% interrater‐agreement), data extraction, and risk of bias assessment with JE. In case of disagreement, we consulted with one of the senior researchers (MdW or KT) until the conflict could be resolved. See Figure 1 for an overview of the screening and inclusion procedure according to the PRISMA guidelines (Matthew et al. 2021).

### 2.3 Data Extraction

We extracted the following data from the articles: (1) name of the first author and year of publication, (2) rate of revictimisation (%), (3) type of revictimisation (sexual, physical, emotional, IPV, or ‘combined’ when measures included various types of revictimisation incidents), (4) the mean score and standard deviation of PTSD symptoms for the sample, (5) measurement instrument for PTSD symptoms, (6) self‐report or clinician‐assessment of PTSD symptoms, (7) type of sample (convenience; veterans; other [see description below]), (8) mean age of the sample, (9) proportion of females in the sample (0–1), (10) sample size, (11) duration of follow‐up period (months), (12) country the study was conducted in, (13) definition of revictimisation/measurement method. Data were extracted by two independent coders (CK and JE), extracted values were then compared. In case of disagreement between the coders, one of the other authors was consulted (KT or MdW). When studies reported separate statistics for different types of revictimisation incidents, we chose the highest estimate since any other strategy would lead to an underestimation of the rate of revictimisation. When a study reported on an overall rate of revictimisation that included different types of revictimisation incidents as well as separate rates of revictimisation per type of violence, we chose the overall rate of revictimisation for the quantitative analysis. When studies had more than two assessments, we chose the assessment at which revictimisation was assessed. If a study had multiple assessments for revictimisation, we chose the one with the longest follow‐up period to decrease the chance of an underestimation of revictimisation. We reported the follow‐up period that corresponded to the assessment of the revictimisation used in the analysis. We contacted authors whenever their articles met all inclusion criteria except providing data for the rate of revictimisation and/or the mean score of PTSD symptoms. We also enquired whether authors could provide subgroup‐statistics whenever the overall sample did not meet all inclusion criteria, but a subgroup did. The following authors provided data via personal communication with us (Scoglio et al. 2022; Cole et al. 2008; Dardis et al. 2018; Iverson et al. 2013; Krause et al. 2006; Lowe et al. 2014; Kiefer et al. 2024). Therefore, some of the numbers presented in figures/tables in this article may deviate slightly from those of the original articles.

### 2.4 Study Quality

We used the risk of bias assessment tool for the prevalence of mental health disorders (RoB‐PrevMH) to assess the risk of bias in our included studies. The RoB‐PrevMH has recently been developed specifically for prevalence studies in the field of mental health (Tonia et al. 2023). Two independent assessors (CK and JE) used the RoB‐PrevMH to assess the included studies along the dimensions of (1) representativeness of the sampling frame (sampling frame bias), (2) representativeness of the responders (responder bias), (3) measurement of the condition (information bias). No overall score is computed. In case of disagreement between the assessors, one of the other authors (MdW or KT) was consulted.

### 2.5 Statistical Analyses

All analyses were conducted using the dmetar and metafor packages in R (version 4.4.1), closely following the step‐by‐step procedure for doing meta‐analysis (and meta‐regression) in R (Harrer et al. 2021). Due to our anticipation of considerable between‐study heterogeneity, we employed random‐effects models and applied the Knapp‐Hartung adjustments. We used the restricted maximum likelihood estimator (RMLE) in our estimation of tau^2^. Given that our outcome was the rate of revictimisation, we used the metaprop function.

### 2.6 Heterogeneity

We used Higgins and Thompsons *I* ^*2*^ statistic to assess between‐study heterogeneity in effect sizes (proportion of revictimised participants in each sample). For the *I* ^*2*^ statistic, 25%, 50%, and > 75% indicate mild, moderate, and substantial heterogeneity, respectively (Higgins and Thompson 2002). In combination with the *I* ^*2*^ statistic, we also report the tau^2^ statistic as an indicator of between‐study heterogeneity that is independent of the number of studies included.

### 2.7 Sensitivity and Subgroup Analysis

We conducted post‐hoc sensitivity analyses with outliers removed from the overall dataset. We further conducted three subgroup analyses with the studies that reported rates of IPV revictimisation, sexual revictimisation, and combined revictimisation. We chose for the outlier‐removal given the large degree of between‐study heterogeneity, with the aim of reducing this heterogeneity. We conducted subgroup analyses to understand the rate of revictimisation for the different types of interpersonal violence, but this was only possible whenever *n* > 2 studies provided revictimisation estimates for that type of revictimisation.

### 2.8 Meta‐Regression

As stated in our pre‐registration, we planned to conduct meta‐regression analyses to identify the univariate effects of (a) the severity of PTSD symptoms in each sample, (b) self‐administered versus clinician‐assessed PTSD measurement, (c) type of sample (convenience, veteran, other), (d) proportion of females in the sample, (e) country the study was conducted in, on the observed variability in rates of revictimisation. However, the diversity in measurement instruments and the lack of population‐based data led us to examine whether the PTSD mean score of each sample lied above/below the clinical cut‐off for the measurement instrument they used (*0* = below clinical cut‐off, *1* = above clinical cut‐off). For the two studies that reported whether participants met diagnostic criteria for PTSD, we coded *0* = less than 50% of the sample met criteria for PTSD and *1* = 50% or more of the sample met criteria for PTSD (Kiefer et al. 2024; Stockdale et al. 2014). Moreover, only *n* = 1 of the included studies reported data for the separate symptom clusters in addition to the overall PTSD mean score (Babcock and DePrince 2013). Therefore, we could not examine the effects of PTSD symptom clusters. Finally, in the process of data‐extraction we became interested in exploring the effects of the duration of the follow‐up period, so we conducted an additional exploratory meta‐regression analysis. All meta‐regression analyses were univariate analyses in which we only examined the effect of one predictor on the observed variance in rates of revictimisation.

### 2.9 Publication Bias

**FIGURE 2:**
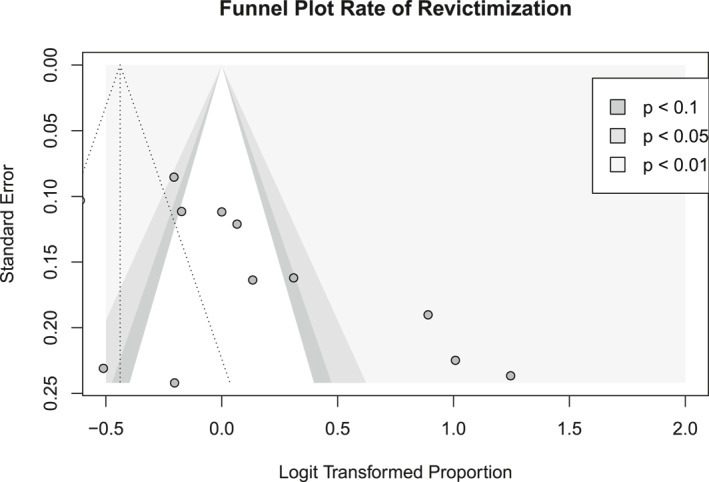
Funnel‐plot of the *N* = 19 included studies.

We visually inspected the Funnel‐plots and conducted Egger's test of the intercept to infer the potential of publication bias, see Figure 2.

## 3 Results

### 3.1 Study Characteristics

We included *N* = 19 studies with a total of *n* = 5661 participants. Sample sizes ranged from *n* = 69 to *n* = 733 participants. Of all included studies, *n* = 16 of 19 studies included only female samples and *n* = 16 of 19 studies were conducted in the U.S. The most prevalent type of revictimisation was IPV (*n* = 11), followed by sexual assault (*n* = 4), mixed types of revictimisation (*n* = 3), and physical revictimisation (*n* = 1). Importantly, measures of IPV commonly consist of a psychological, physical, and sexual abuse scale (e.g., Conflict‐Tactics Scale; M. A. Straus et al. 1996). Study populations were predominantly convenience samples (i.e., individuals who reacted to advertisements in local papers/at college/online; *n* = 6), veteran samples (*n* = 2), and ‘other’ samples (i.e., some were help‐ or compensation‐seeking survivors of violence, battered women staying in shelters, or women recruited after obtaining a violence protection order, *n* = 11), see Table 1 for a description of the sample per study. Rates of revictimisation ranged from 7.6% (Stockdale et al. 2014) to 78.0% (S. Perez et al. 2012). The follow‐up periods of studies ranged from 1–276 months, with most studies (84.2%) employing a follow‐up period between 6 and 24 months. Roughly two‐thirds (63.2%; *n* = 12) of the included studies assessed the PTSD symptoms via self‐report. See Table 1 for an overview of study characteristics and definitions of revictimisation and Table 2 for the data we extracted.

**TABLE 1:** Study characteristics and definitions of revictimisation of the *N* = 19 included studies.

Authors, year	Sample description	Type(s) of revictimisation (%)	Definition of revictimisation	Measurement instrument revictimisation	Measurement instrument PTSD symptoms	Location
Banyard et al. 2002	Female survivors of childhood sexual abuse	^1^Physical revictimisation (37.5%), sexual revictimisation (27.6%)	Participants were asked which of the following they experienced in the past 276 months: Attacked with and without a weapon, sexually assaulted by a stranger, sexually assaulted by someone known to them, sexually assaulted with the use of force or threat of force.	Questions adapted from a measure in the national women's study (Resnick et al. 1996)	Trauma symptom checklist (TSC 40; Elliott and Briere 1992)	USA
Bell et al. 2008	Help‐seeking female survivors of intimate partner violence (IPV)	Psychological IPV revictimisation (52%)	Participants answered two questions about whether they experienced dominance/isolation or emotional/verbal abuse from an intimate partner in the past 18 months.	Psychological maltreatment of women inventory ‐ short form (Tolman 1999)	PTSD checklist specific IV (PCL‐S‐IV; Blanchard et al. 1996)	USA
Cascardi 2016	Participants were involved with the child protective services for being alleged victims of child abuse/neglect. Only those who were older than 18 and female were selected for the study.	Physical IPV revictimisation (27.4%)	The total number of violent acts experienced in the past 12 months as indicated on their measure of revictimisation.	Physical violence subscale of the conflict tactics scale—Revised (CTS‐R; M. A. Straus et al. 1996)	Trauma symptom checklist for children—10 item version (TSC 10; Briere 1996)	USA
Cole et al. 2008	Female survivors of IPV, that had recently obtained a domestic violence order (DVO) against an intimate partner	IPV revictimisation (35.2%)	Scoring a ‘yes’ on any of their measures for revictimisation that arses the physical, psychological and sexual dimensions of intimate partner violence in the past 12 months.	Questions adapted from other studies on protective orders (Harrell et al. 1993), from the CTS and CTS‐R (M. A. Straus et al. 1996; M. Straus 1995), and Toman's psychological maltreatment of women inventory (PMWI; Tolman 1999)	Diagnostic interview schedule (DIS; Robins et al. 1981)	USA
Dardis et al. 2018	Female veterans that reported past‐year IPV and were part of the knowledge panel, a probability‐based access survey panel	IPV revictimisation (*73.3%)	Scoring on any of the subscales of the revictimisation measure. Thus, experiencing any type of IPV in the past 6 months.	CTS‐R (M. A. Straus et al. 1996)	PTSD checklist −5 (PCL‐5; Blevins et al. 2015)	USA
Dokkedahl et al. 2022	Women staying at battered women's’ shelters	IPV revictimisation 53.6%	Participants were first asked whether they experienced a new episode of violence in the past 12 months, consequently they were asked to specify whether they experienced: Physical, psychological, sexual, economic, or honour‐related violence in the past 12 months.	Dichotomous measure that was designed by the authors themselves.	International trauma questionnaire (ITQ; Cloitre et al. 2021)	Denmark
Iverson et al. 2013	Help‐seeking female IPV survivors whose most recent IPV episode was no longer than 2–6 months ago	Physical IPV revictimisation (46.0%)	Participants were asked whether they had experienced physical IPV by a former or current partner in the past 6 months.	Physical assault subscale of the CTS‐R (M. A. Straus et al. 1996)	Posttraumatic diagnostic scale (PDS; Foa et al. 1997)	USA
Kiefer et al. 2024	Female IPV survivors from the community	IPV revictimisation (*71%)	Participants received daily questions concerning the occurrence of any IPV (yes/no) since the day before. The rate of revictimisation is based on how many participants indicated any new incident of IPV during the study period (1 month).	Three dichotomous (yes/no) questions regarding the occurrence of physical, psychological, and/or sexual IPV.	Structured clinical interview for DSM‐5 (SCID‐5; First 2014)	USA
Krause et al. 2006	Help‐seeking female IPV survivors	Physical and/or sexual IPV revictimisation (45.9%)	Participants were asked whether they had experienced a new episode of physical and/or sexual violence by their index partner (who had been violent prior to participant's inclusion in the study) in the past 12 months.	Physical and sexual assault subscales of the CTS‐R (M. A. Straus et al. 1996)	Post traumatic symptom checklist IV (PCL‐IV; Blanchard et al. 1996)	USA
Kuijpers et al. 2012	Help seeking female IPV survivors	^1^Psychological IPV revictimisation (58.3%), physical IPV revictimisation (25.6%)	Participants were asked whether they experienced psychological or physical IPV in the past 2 months (yes/no).	Dichotomous version of the psychological and physical assault subscales of the CTS‐R (M. A. Straus et al. 1996)	PTSD symptom scale ‐self ‐report version (PSS‐SR; Foa et al. 1993)	Netherlands
Kunst et al. 2010	Survivors of interpersonal violence, seeking compensation from the Dutch victim compensation fund	^1^Cumulative revictimisation (22.8%), physical assault (3.5%), threats of violence (9.4%), sexual assault (9.9%)	Participants were asked whether they had experienced sexual harassment, threat of physical violence, or physical violence in the past 6 months.	Dutch safety survey (veiligheidsmonitor; CBvd 2008)	PSS‐SR (Foa et al. 1993)	Netherlands
Littleton et al. 2009	Female college students that indicated a history of sexual violence	Sexual revictimisation: ^1^completed rape (30%), attempted rape (30%)	Participants were asked about experiences of rape or sexual assault in the past 6 months, defined as unwanted sex with a man or men (vaginal, oral, anal intercourse, or object penetration) obtained by force or threat of force, or that occurred when the individual was incapacitated or unconscious.	Two screening items that were derived from the sexual experiences survey (SES; Koss and Gidycz 1985)	PSS‐SR (Foa et al. 1993)	USA
Littleton and Decker 2017	Female college students that indicated a history of sexual violence	Sexual revictimisation (16.3%)	Participants were asked whether they had experienced unwanted touching, attempted rape, and completed rape in the past 2 months.	Nine items from the sexual experiences survey—revised (SES‐R; Koss et al. 2007)	PSS‐SR (Foa et al. 1993)	USA
Lowe et al. 2014	Predominantly non‐hispanic black residents of urban Detroit that indicated exposure to previous interpersonal trauma	Cumulative revictimisation (*26.2%)	Participants were asked whether they experienced ‘assaultive trauma’ in the past 12 months.	The eight assaultive trauma items of the 20‐item trauma inventory of lifetime traumatic events (Breslau et al. 1998)	Posttraumatic symptom checklist—civilian IV (PCL‐C‐IV; Blanchard et al. 1996)	USA
Najdowski and Ullman 2009	Adult women with unwanted sexual experiences since age 14, that responded to recruitment efforts in the community/the newspaper	Sexual assault (45.0%)	Participants were asked about any new experience of completed rape, attempted rape, sexual coercion, or unwanted sexual contact in the past 12 months. A positive answer to any of those prompts was counted as ‘revictimised’.	A dichotomous version of the SES (Koss and Gidycz 1985)	PDS (Foa et al. 1997)	USA
S. Perez et al. 2012	Female residents of one of two battered women's shelters	IPV revictimisation (78.0%)	Participants were asked about any new experience of intimate partner violence in the past 6 months.	CTS‐R (M. A. Straus et al. 1996)	Clinician administered PTSD scale for DSM‐IV (CAPS‐IV; Blake et al. 1995)	USA
S. K. Perez and Johnson 2008	Female survivors of IPV who were recruited at hospitals and medical clinics	Physical IPV revictimisation (50%)	Participants were asked about any experiences of violence during the past 12 months.	Participants were provided with a blank calendar, representing the past year. They filled in important events, such as a new job, etc, together with a researcher. Subsequently they added violent incidents, if they experienced any.	PTSD symptom scale interview (PSS‐I; Foa et al. 1993)	USA
Scoglio et al. 2022	Veterans who were survivors of childhood abuse and/or military sexual trauma	Sexual and/or physical revictimisation (*13.2%)	Participants were asked about any experience of unwanted sexual activity as a result of force, threat of harm, or manipulation and/or having been seriously physically injured by another person in the past 12 months.	Two items from the deployment risk and Resilience inventory (DRRI; King et al. 2006)	PCL‐C‐IV (Blanchard et al. 1996)	USA
Stockdale et al. 2014	Female IPV survivors who have recently requested a DVO against a previous intimate partner	Sexual revictimisation (7.6%)	Participants were asked about any experience where a colleague/supervisor made them feel like they had to ‘do something sexual’ in the past 12 months.	An adapted version of the sexual experiences questionnaire (SEQ‐DoD; Fitzgerald et al. 1999)	DIS (Robins et al. 1981)	USA

*Note:* For the studies reporting multiple rates of revictimisation, we used subscript 1 to indicate the rate that was used in the analysis. Information marked with * is based on personal communication with the authors and thus deviates from the referenced article.

**TABLE 2:** Data extracted from the *N* = 19 included studies.

Authors, year of publication	Rate of revictimisation (%)	Severity of PTSD *M* (SD)	Presence of severe PTSD symptoms	Mode of assessment	Type of sample	Proportion of females in sample (0–1)	Duration follow‐up period (Months)	Sample size	Mean age sample
Banyard et al. 2002	37.5	27.48 (21.82)	0	0	1	1	276	80	25.5
Bell et al. 2008	52.0	47.55 (18.17)	1	1	3	1	18	273	33
Cascardi 2016	27.4	47.70 (8.75)	0	1	1	1	96	532	12.8
Cole et al. 2008	35.2	*7.45 (*5.11)	0	0	3	1	12	412	31.7
Dardis et al. 2018	*73.3	*14.46 (*18.35)	0	1	2	1	6	101	49.4
Dokkedahl et al. 2022	53.6	15.30 (15.40)	1	1	3	1	12	150	34.6
Iverson et al. 2013	46.0	*29.42 (*10.71)	1	1	3	1	6	69	35.9
Kiefer et al. 2024	*71.0	(38% of the sample had PTSD)	0	0	3	1	1	134	40.7
Krause et al. 2006	45.9	*48.15 (*18.51)	1	1	3	1	12	324	33.0
Kuijpers et al. 2012	58.3	20.21 (4.91)	1	1	3	1	6	156	37.7
Kunst et al. 2010	22.8	15.7 (12.75)	1	1	3	0.62	6	202	44.2
Littleton et al. 2009	30.0	10.54 (9.87)	0	1	1	1	6	334	21.7
Littleton and Decker 2017	16.3	10.38 (10.86)	0	1	1	1	2	206	20.9
Lowe et al. 2014	*26.2	*34.85 (*15.25)	0	0	1	0.57	24	733	50.6
Najdowski and Ullman 2009	45.0	19.06 (12.38)	1	1	1	1	12	555	33.0
S. Perez et al. 2012	78.0	54.12 (27.34)	1	0	3	1	6	103	35.5
S. K. Perez and Johnson 2008	50.0	10.23 (5.13)	0	0	3	1	12	320	31.0
Scoglio et al. 2022	*13.2	*49.25 (*17.46)	1	1	2	0.46	12	532	35.6
Stockdale et al. 2014	7.6	9.91 (3.63)	0	0	3	1	12	445	31.9

*Note:* Information marked with * is based on personal communication with the authors and thus deviates from the referenced article. The mean age of the sample and the sample size refer to the assessment during which the PTSD symptoms were measured, which preceded (sometimes substantially) the measurement of revictimisation.

Abbreviations: *M* = mean, SD = standard deviation, PTSD = post‐traumatic stress disorder. We coded such that for presence of severe PTSD symptoms: 0 = absent, 1 = present; for mode of assessment of PTSD symptoms: 0 = clinician‐assessed, 1 = self‐report; for type of sample: 1 = convenience, 2 = veterans, 3 = other.

### 3.2 Study Quality

**FIGURE 3:**
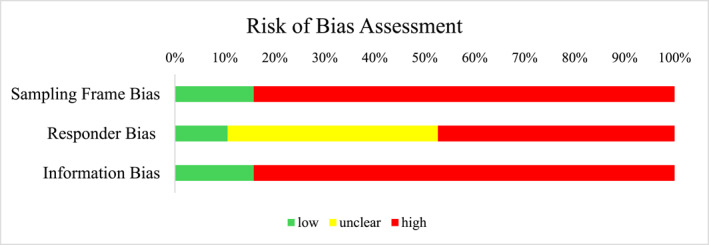
Overview of risk of bias assessment with the RoB‐PrevMH for *N* = 19 studies.

Figure 3 shows that of the *N* = 19 included studies, 84% (*n* = 16) scored high on information bias (i.e., how the outcome [revictimisation] was measured) and sampling frame bias (i.e., were those invited representative of the target population), respectively. Just under half of the studies 47.0% (*n* = 9) scored high on responder bias (i.e., high incidence of non‐response or non‐response due to systematic differences between responders/non‐responders). In total, *n* = 4 studies scored high on all three dimensions of bias. See Supporting Information S3: Appendix 3 for an overview of risk of bias ratings per study.

### 3.3 Meta‐Analysis on the Rate of Revictimisation

**FIGURE 4:**
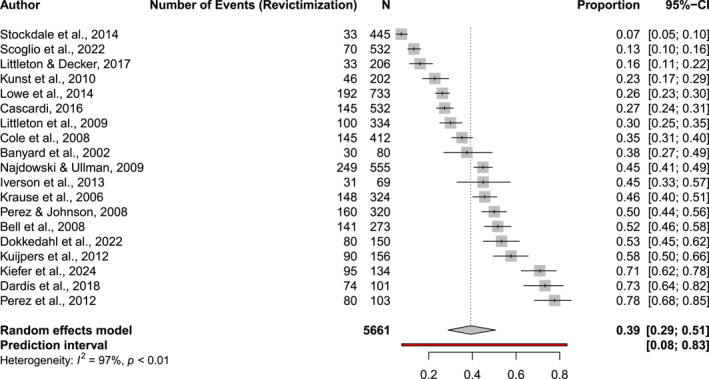
Forest‐plot for the meta‐analysis of *N* = 19 studies.

**FIGURE 5:**
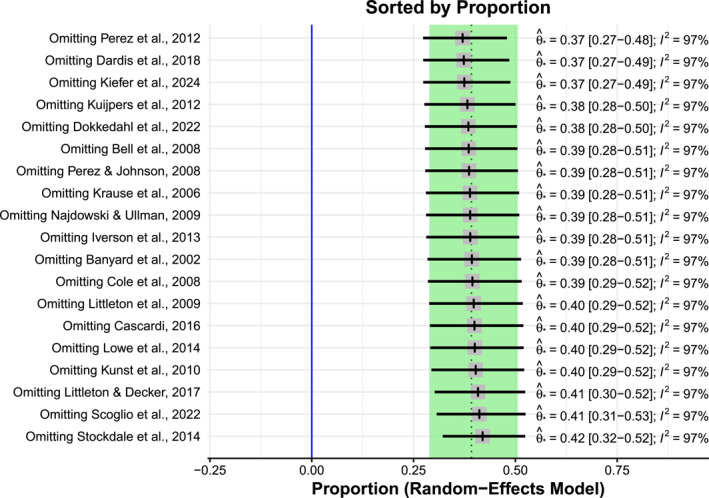
Leave‐one‐out analysis for the *N* = 19 included studies.

The main meta‐analysis of this study showed that the pooled rate of revictimisation across all *N* = 19 studies was 39.2% (CI_95_[28.9, 50.6]). Between‐study heterogeneity in effect sizes was substantial with *I* ^*2*^ = 97.1%, CI_95_[96.4, 97.7], *p* < 0.001, and tau^2^ = 0.90. For a forest plot of the effect sizes, see Figure 4. The visual inspection of the funnel plot (Figure 2) suggests that there may be publication bias. In the case of no publication bias, one would expect a symmetric distribution of studies (individual points) within the triangle that represents the 95% confidence interval of the pooled effect size (rate of revictimisation). In the current funnel plot (Figure 2) there was asymmetry in the distribution of the studies as well as three outliers. Therefore, the funnel plot strongly suggests that publication bias may lead to an overestimation of the true effect size. At the same time, Egger's test of the intercept does not imply a potential for publication bias (*t* = 0.82, *p* = 0.423). Given the large degree of between‐study heterogeneity as indicated by *I* ^*2*^, we decided to also include a plot of the leave‐one‐out analysis, see Figure 5. The leave‐one‐out analysis indicates the change in effect size for omitting each study individually. We saw no big changes if we omitted any one study, indicating that there was not a single study that had a substantial impact on the pooled rate of revictimisation.

### 3.4 Sensitivity Analysis With Outliers Removed

We conducted a sensitivity analysis in which we removed the outliers in R via the find.outliers function (*metafor* package). This led to a pooled rate of revictimisation of 39.8% (CI_95_[33.1, 47.0]) for *n* = 13 studies (Najdowski and Ullman 2009; Dokkedahl et al. 2022; Cole et al. 2008; Iverson et al. 2013; Krause et al. 2006; Lowe et al. 2014; Banyard et al. 2002; Bell et al. 2008; Cascardi 2016; Littleton et al. 2009; S. K. Perez and Johnson 2008; Kunst et al. 2010; Kuijpers et al. 2012). Heterogeneity was still substantial with *I* ^*2*^ = 93.9%, CI_95_[91.2, 95.8], *p* < 0.001, and tau^2^ = 0.21. The rate of revictimisation for the sensitivity analysis was comparable with that of the main analysis (39.8% and 39.2%, respectively). Additionally, based on two substantial outliers with regard to the length of the FU‐period we also conducted a sensitivity analysis without these two studies (Banyard et al. 2002; Cascardi 2016). This led to a pooled rate of revictimisation of 40.1% (CI_95_[28.5, 52.9]). Heterogeneity was substantial with *I* ^*2*^ = 97.4%, CI_95_[96.6, 97.9], *p* < 0.001, and tau^2^ = 0.97. In sum, the rate of revictimisation for this sensitivity analysis was comparable to the main analysis (40.1% vs. 39.2%, respectively).

### 3.5 Subgroup Analyses

#### 3.5.1 Intimate Partner Violence Revictimisation

We decided post‐hoc to repeat the procedure of the main analysis for the *n* = 11 studies that reported IPV revictimisation (Dokkedahl et al. 2022; Cole et al. 2008; Dardis et al. 2018; Iverson et al. 2013; Krause et al. 2006; Kiefer et al. 2024; S. Perez et al. 2012; Bell et al. 2008; Cascardi 2016; S. K. Perez and Johnson 2008; Kuijpers et al. 2012). The pooled rate of revictimisation was 53.4% CI_95_[42.6, 63.9], which was higher than that for the overall group 39.2% (CI_95_ [28.9, 50.6], *N* = 19), although this difference was not statistically significant. Heterogeneity was again substantial with *I* ^*2*^ = 95.0%, CI_95_[92.7, 96.6], *p* < 0.001, and tau^2^ = 0.39.

#### 3.5.2 Sexual Revictimisation

We also repeated the procedure of the main analysis for the *n* = 4 studies that provided primary estimates on sexual revictimisation (Najdowski and Ullman 2009; Stockdale et al. 2014; Littleton et al. 2009; Littleton and Decker 2017). The pooled rate of sexual revictimisation was 21.4% CI_95_[0.1, 52.3], a lower estimate than that for the overall group, 39.2% (CI_95_ [28.9, 50.6], *N* = 19), although this difference was not statistically significant. Heterogeneity was substantial with *I* ^*2*^ = 98.1%, CI_95_[96.9, 98.9], *p* < 0.001, and tau^2^ = 0.75.

#### 3.5.3 Combined Revictimisation

We also pooled the rate of revictimisation for all *n* = 3 studies that reported a combined estimate of revictimisation that spans multiple types of violence, that is, physical and sexual violence that were perpetrated by someone who was not necessarily and intimate partner (Scoglio et al. 2022; Lowe et al. 2014; Kunst et al. 2010). The pooled rate of combined revictimisation was 20.1% CI_95_[0.1, 38.9], which was lower than the estimate for the overall group, 39.2% (CI_95_ [28.9, 50.6], *N* = 19), although this difference was not statistically significant. Heterogeneity was substantial with *I* ^*2*^ = 93.5%, CI_95_[84.5, 97.3], *p* < 0.001, and tau^2^ = 0.12.

### 3.6 Meta‐Regression

The meta‐regression on the effect of (a) the presence of severe PTSD (using the absence as the reference category) led to a non‐significant model (*β* = 0.41, *p* = 0.352). Therefore, the presence of severe PTSD symptoms does not significantly account for the observed variability in rates of revictimisation. See Table 3 for an overview of all reported meta‐regression analyses.

**TABLE 3:** Overview of predictors in the univariate meta‐regression analyses (*N* = 19).

Variable	Samples	*β*	CI_95_ LL‐UL	*p*‐value	QM (test of moderators)	Heterogeneity
Presence of severe PTSD						
Present	9	0.41	−0.50–1.32	0.352	0.92	*Tau* ^*2*^ = 0.85
						*I* ^*2*^ = 97.9%
						*H* ^*2*^ = 48.3
Absent	10	Reference category				
Mode of assessment (PTSD)						
Self‐report	12	−0.11	−1.07–0.86	0.820	0.05	*Tau* ^*2*^ = 0.89
						*I* ^*2*^ = 98.0%
						*H* ^*2*^ = 50.3
Clinician‐assessed	7	Reference category				
Type of sample						
Veterans	2	−0.03	−1.55–1.49	0.970	0.00	*Tau* ^*2*^ = 0.90
						*I* ^*2*^ = 98.1%
						*H* ^*2*^ = 52.2
‘Other’	11	0.58	−0.32–1.48	0.189	1.87	*Tau* ^*2*^ = 0.81
						*I* ^*2*^ = 97.8%
						*H* ^*2*^ = 46.0
Convenience	6	Reference category				
Proportion of females	19	2.56	0.07–5.04	0.044	4.72	*Tau* ^*2*^ = 0.71
						*I* ^*2*^ = 97.5%
						*H* ^*2*^ = 39.8
Location						
Europe	3	0.21	−1.06–1.48	0.729	0.12	*Tau* ^*2*^ = 0.89
						*I* ^*2*^ = 98.1%
						*H* ^*2*^ = 52.4
U.S.	16	Reference category				
Duration FU‐period	19	−0.00	−0.01–0.01	0.730	0.13	*Tau* ^*2*^ = 0.89
						*I* ^*2*^ = 98.1%
						*H* ^*2*^ = 52.1

Abbreviations: CI_95_ LL‐UL = upper and lower limit of the 95% confidence interval; FU‐period = follow‐up period; QM = moderator test (analogue to the *F*‐statistic).

Next, we conducted a meta‐regression analysis on the effect of (b) assessing PTSD symptoms via self‐report or interview by a clinician, using clinician‐assessed symptoms as the reference category. The effects of self‐report assessment were non‐significant (*β* = − 0.11, *p* = 0.820). Therefore, the mode of assessment of PTSD symptoms did not significantly account for the observed variability in rates of revictimisation.

We conducted two meta‐regression analyses on the effects of (c) the type of sample under investigation, with the category ‘convenience’ as the reference category and dummy variables for ‘veterans’ and ‘other’. The effect for veterans (*β* = −0.03, *p* = 0.970) and for ‘other’ samples (*β* = 0.58, *p* = 0.189) were non‐significant. Therefore, the type of sample did not significantly account for the observed variability in rates of revictimisation.

We conducted a meta‐regression on the effect of (d) the proportion of females in the sample (0–1), which was significant (*β* = 2.56, *p* = 0.044). Therefore, the proportion of females in the sample did account for some of the observed variability in rates of revictimisation.

Next, we conducted a meta‐regression on the effect of (e) the country the study was conducted (Europe vs. U.S.), with the U.S. as the reference category, which was non‐significant (*β* = 0.21, *p* = 0.729). Therefore, the country in which the study was conducted did not significantly account for the observed variability in rates of revictimisation.

Finally, we conducted an exploratory meta‐regression on (f) the effect of the duration of the follow‐up period, which was also non‐significant (*β* = −0.00, *p* = 0.730). Therefore, the duration of the follow‐up period did not significantly account for the observed variability in rates of revictimisation.

We had planned to conduct a multivariate meta‐regression with all the effects that were significant in the univariate analyses. However, due to only one significant univariate effect (proportion of females in the sample), we could not proceed with a multivariate model.

### 3.7 Exploratory Meta‐Regression in the IPV Revictimisation Subgroup

In addition to our pre‐registered analyses, we investigated the same univariate regression effects in the IPV revictimisation subgroup. This decision was based on the lower rate of heterogeneity and the high rate of revictimisation, underscoring the necessity of understanding risk factors for revictimisation in this particular group. We could not conduct the meta‐regression on the effect of proportion of females in the sample, since all of the IPV revictimisation studies were conducted in all‐female samples.

The meta‐regression on the effect of the presence of severe PTSD (using the absence as the reference category) led to a non‐significant model (*β* = 0. 19, *p* = 0.638), see Table 4 for an overview of all reported meta‐regression analyses. The effects of self‐report assessment (of PTSD symptoms) were non‐significant (*β* = −0.34, *p* = 0.406), using clinician‐assessment as the reference category. The effect for veterans (*β* = 0.97, *p* = 0.162) and for ‘other’ samples (*β* = 0.21, *p* = 0.691) were non‐significant, using convenience samples as the reference category. Next, we conducted a meta‐regression on the effect of the location (Europe vs. U.S.), with the U.S. as the reference category, which was non‐significant (*β* = 0.11, *p* = 0.839). Finally, the effect of the duration of the follow‐up period was the only significant predictor, (*β* = −0.02, *p* = 0.020), whereby a shorter FU period was associated with a higher rate of revictimisation.

**TABLE 4:** Overview of predictors in the exploratory meta‐regression analyses for the IPV revictimisation subgroup (*n* = 11).

Variable	Samples	*β*	CI_95_ LL‐UL	*p*‐value	QM (test of moderators)	Heterogeneity
Presence of severe PTSD						
Present	6	0.18	−0.69–1.06	0.638	0.24	*Tau* ^*2*^ = 0.38
						*I* ^*2*^ = 95.0%
						*H* ^*2*^ = 19.8
Absent	5	Reference category				
Mode of assessment (PTSD)						
Self‐report	7	−0.34	−1.24–0.55	0.406	0.76	*Tau* ^*2*^ = 0.37
						*I* ^*2*^ = 94.7%
						*H* ^*2*^ = 18.9
Clinician‐assessed	4	Reference category				
Type of sample						
Veterans	1	0.97	−0.47–2.41	0.162	2.32	*Tau* ^*2*^ = 0.32
						*I* ^*2*^ = 94.6%
						*H* ^*2*^ = 18.4
‘Other’	8	0.21	−0.93–1.34	0.691	0.17	*Tau* ^*2*^ = 0.38
						*I* ^*2*^ = 94.8%
						*H* ^*2*^ = 19.2
Convenience	2	Reference category				
Location						
Europe	2	0.11	−1.04–1.25	0.839	0.04	*Tau* ^*2*^ = 0.39
						*I* ^*2*^ = 95.3%
						*H* ^*2*^ = 21.4
U.S.	9	Reference category				
Duration FU‐period	11	−0.02	−0.03–0.00	0.020*	7.90	*Tau* ^*2*^ = 0.21
						*I* ^*2*^ = 90.8%
						*H* ^*2*^ = 10.9

*Note:* Statistically significant results at *p* < 0.05 are indicated with *.

Abbreviations: CI_95_ LL‐UL = upper and lower limit of the 95% confidence interval; FU‐period = follow‐up period; QM = moderator test (analogue to the *F*‐statistic).

## 4 Discussion

The current study is a meta‐analysis and meta‐regression on *N* = 19 longitudinal studies reporting on revictimisation and PTSD symptoms. We have computed a pooled rate of revictimisation of 39.2% for the overall group, 53.4% for the IPV revictimisation, 21.4% for the sexual revictimisation group, and 20.1% for the combined revictimisation group. The latter two estimates were much lower than was expected based on previous literature and had remarkably wide confidence intervals. We examined the effects of study characteristics on the rates of revictimisation across studies. The presence of severe PTSD symptoms, mode of assessment of PTSD symptoms, type of sample, location, and duration of the follow‐up period were not associated with rates of revictimisation. The proportion of females was positively associated with the rate of revictimisation, indicating that the higher the proportion of females in the sample, the higher the rate of revictimisation. Exploratory meta‐regression analyses of studies on IPV revictimisation revealed that shorter follow‐up periods were associated with higher rates of revictimisation in this subgroup. Previous meta‐analyses on revictimisation have provided similar estimates, with Walker et al. (2019) reporting that 47.9% of all CSA survivors experienced sexual revictimisation in adulthood. Throughout this discussion, we discuss the strengths and limitations within each section to place them in context. One general limitation that applies to all sections is that the two ways in which we assessed the potential for publication bias (Funnel plot and Egger's test of the intercept) were contradictory. Therefore, we cannot exclude the possibility that publication bias may have led to an overestimation of the effect sizes (rates of revictimisation).

Concerning the pooled rate of revictimisation that we have computed, we need to stress that this is the result of an analysis that indicated a substantial degree of heterogeneity in observed effect sizes (*I* ^*2*^). This is an important note of caution in the interpretation of the pooled rate of revictimisation since this seems to indicate the possibility that there were multiple *true effect sizes* in our dataset (Harrer et al. 2021; Borenstein et al. 2017). We may have included multiple true effect sizes that were specific to certain types of revictimisation incidents or samples. A potential consequence of multiple true effect sizes could be that pooling these rates of revictimisation leads to a distorted estimate due to overlapping confidence intervals of the separate true effect sizes. Supporting our notion that we potentially have multiple effect sizes in our dataset is the wide range of revictimisation rates (7.6%–78.0%; Stockdale et al. 2014; S. Perez et al. 2012). While we still fully support the idea of calculating an umbrella‐statistic for the rate of revictimisation, we also call for more uniformity between studies in the definition of revictimisation (among others).

### 4.1 Subgroup Analyses

In the subgroup analysis, we found a higher rate of revictimisation for the IPV revictimisation group than for the overall group (53.4 vs. 39.25%, respectively). A recent meta‐analysis on risk factors for IPV revictimisation did not provide a pooled rate of revictimisation but indicated that the rate of revictimisation ranged between 23.7%–50.5% in the subset of longitudinal studies (Bellot et al. 2024). This may indicate that those experiencing IPV victimization are at higher risk of revictimisation. Or it may indicate that the studies on the other types of revictimisation (sexual, cumulative, and physical revictimisation) may be even more heterogeneous (contain multiple true effect sizes), undermining our ability to compute a meaningful rate of revictimisation. Taking a closer look at the second to largest group in the dataset (sexual revictimisation) illustrates this point, since the definitions of sexual revictimisation and measurement methods ranged widely between studies. This may partially explain our unexpectedly low estimate of sexual revictimisation with its corresponding extremely wide confidence interval (21.4%, CI_95_[0.1, 52.3]). The context in which sexual revictimisation was investigated ranged from the military context to the workplace, to sexual revictimisation in college (Scoglio et al. 2022; Stockdale et al. 2014; Littleton et al. 2009). Similarly, the definition of sexual revictimisation also differed greatly, with some studies defining sexual revictimisation as being afraid to be reprimanded if one would not ‘do something sexual’ for a supervisor or colleague (Stockdale et al. 2014) to ‘completed rape’ which referred to unwanted oral, anal or vaginal penetration, when incapacitated (Littleton et al. 2009). This range of definitions reinforces our notion of the potential for multiple true effect sizes in our sample.

Regarding our third subgroup analysis of the combined types of interpersonal violence (not exclusively perpetrated by an intimate partner), the same observation as for the sexual revictimisation holds. The estimated rate of revictimisation was much lower than anticipated and had an extremely wide confidence interval (20.1% CI_95_[0.1, 38.9]). We also noticed that many of the studies that assessed both sexual revictimisation and IPV revictimisation used ‘adapted versions’ or subscales of validated questionnaires (Cole et al. 2008; Iverson et al. 2013; Stockdale et al. 2014; Littleton et al. 2009; S. K. Perez and Johnson 2008) or they simply asked (yes/no) whether participants experienced certain types of revictimisation (Scoglio et al. 2022; Dokkedahl et al. 2022; Kiefer et al. 2024). The lack of clear operationalisation and standardized/validated measurement instruments also became apparent in the risk of bias assessment, where 84.0% of included studies had a high risk of information bias due to questionable assessment of the rate of revictimisation. The subgroup of IPV revictimisation studies did show the greatest consistency in terms of measurement instruments, since the vast majority used a version/subscale of the Conflict Tactics Scale—Revised (M. A. Straus et al. 1996).

### 4.2 Presence of Severe PTSD Symptoms

Contrary to our hypothesis, we found no effect of the presence of severe PTSD symptoms on variability in rates of revictimisation. This finding is not in line with systematic reviews, which have demonstrated that PTSD symptoms were a risk factor for revictimisation in (a subset of) longitudinal studies (Fereidooni et al. 2023; Kühner et al. Forthcoming). Additionally, a meta‐analysis on IPV revictimisation demonstrated that the severity of PTSD was associated with a higher risk of revictimisation in a subset of longitudinal studies (Bellot et al. 2024). The important difference between the current study and the systematic reviews is that the latter summarise (narrate) findings of individual studies while relying only on the inferential statistics of the original articles. In those cases, the overall severity of PTSD symptoms emerges as one of the most robust predictors of revictimisation across studies (Kühner et al. Forthcoming). Due to the extensive heterogeneity in instruments used to measure PTSD and the lack of population data that would enable us to normalise/standardise PTSD symptoms scores, we had to dichotomise the PTSD symptom severity. Thus, we tested the effects of whether the mean‐score of the sample was above or below the clinical cut‐off for the measurement instrument they used. In doing so, we lost a lot of information, and we cannot exclude the possibility that the effect of the severity of PTSD symptoms may have been lost in the process. On the study‐level there is ample evidence that higher PTSD symptoms are associated with a higher risk for revictimisation (Scoglio et al. 2022; Dardis et al. 2018; Babcock and DePrince 2013; S. Perez et al. 2012; S. K. Perez and Johnson 2008; Iverson et al. 2022). Relatedly, in a study in patients with PTSD and psychotic disorders, the risk of revictimisation was significantly lower for those who had received treatment for PTSD than for those in the waitlist condition (van den Berg et al. 2016). Taken together, these findings may suggest a dose‐response relationship between the severity of PTSD and the risk for revictimisation. We could not accomplish a nuanced analysis of this relationship due to the heterogeneity in PTSD measurement and through dichotomisation may have underestimated the relationship between PTSD symptoms and revictimisation. In sum, conducting nuanced meta‐science on the influence of PTSD symptoms on rates of revictimisation is too difficult in the current body of literature. Therefore, we call for more uniformity in the assessment of PTSD symptoms. This can be achieved by either using the gold‐standard for measuring PTSD symptoms, such as the clinician administered PTSD scale for DSM‐5 (CAPS‐5), and/or the PTSD symptom checklist for DSM‐5 (PCL‐5) for self‐report (Blevins et al. 2015; Weathers et al. 2018). Another route would be the reporting of a common‐metric, such as t‐scores, for PTSD symptoms. This approach has been advocated to allow comparisons across studies (de Beurs et al. 2022).

### 4.3 Mode of Assessment of PTSD Symptoms

We found no effect of the mode of assessment of PTSD symptoms on rates of revictimisation. We had no prior hypothesis about the direction of the effect (or the presence thereof). We noticed that one third of the included studies used an interview‐format to assess PTSD symptoms.

### 4.4 Type of Sample

Contrary to our hypothesis, we found no effect of the type of sample used in a study (convenience, veteran, other) on rates of revictimisation. This is contrary to a meta‐analysis on sexual revictimisation that found an effect for the type when comparing college versus other samples (Roodman and Clum 2001). At the same time, a more comprehensive meta‐analysis on sexual revictimisation found no effect for the type of sample when comparing college versus other samples (Walker et al. 2019). Similar to the aforementioned meta‐analyses, we also encountered some difficulties in coding the ‘convenience/veteran’ samples due to the great heterogeneity in types of samples in this category. This heterogeneous ‘other’ category may have influenced our ability to find an effect of type of sample on the variability in rates of revictimisation. We recognise the importance of testing effects in different samples to extend the validity of our findings to different populations. At the same time, this may be a strength on an individual study basis that turns into a pitfall when doing meta‐science. We call for more clarification on the role that for example PTSD symptoms play in one type of sample, before moving to a different type of sample with an incomplete picture of which factors are predictive of revictimisation.

### 4.5 Proportion of Females in the Sample

We found an effect for the proportion of females in the sample on rates of revictimisation, whereby a higher proportion of females led to a higher rate of revictimisation. At the same time, only three of the nineteen included studies had mixed samples (Scoglio et al. 2022; Lowe et al. 2014; Kunst et al. 2010). Of these three, two still consisted of a majority of women (Lowe et al. 2014; Kunst et al. 2010), underlining a lack of diversity in gender‐composition of samples. We cannot exclude the possibility that the overrepresentation of females may have statistically inflated our power to find an effect for the proportion of females in the sample. Moreover, the three studies that included mixed‐gender samples may have differed in aspects other than gender‐composition from the sixteen female‐only studies. Potentially the types of revictimisation under investigation also contributed to the risk of revictimisation. Namely, in all three mixed‐gender studies the revictimisation estimate was based on a cumulative score that included both physical and sexual revictimisation. This may have contrasted these studies with other studies that investigated sexual or IPV revictimisation. We call for more diversity in the composition of samples in terms of gender and to the inclusion of non‐binary/transgender participants, since gender‐minorities have been demonstrated to be at increased risk of sexual revictimisation (Blackburn et al. 2023).

### 4.6 Location

We found no effect of the location (U.S. vs. Europe) on the rates of revictimisation. At the same time, sixteen of the nineteen included studies were conducted in the U.S. We call for more attention to revictimisation in the context of PTSD in both Europe and other parts of the world.

### 4.7 Duration Follow‐Up Period

We found no effect of the duration of the follow‐up period on the rates of revictimisation. One implication of this finding is that during shorter follow‐up periods, similar rates of revictimisation are observed as during longer follow‐up periods. This is contrary to our expectation, since a longer follow‐up period increases the chances of revictimisation incidents as there is simply more time and thus opportunity for revictimisation to occur. At the same time, most of the studies had follow‐up periods of 6–24 months, with two studies conducting significantly longer follow‐up measurements (96 and 276 months; Banyard et al. 2002; Cascardi 2016) and one study a significantly shorter follow‐up measure of one month (Kiefer et al. 2024). Given the large differences between studies on both, duration of follow‐up periods as well as other methodological aspects, we cannot draw conclusions about whether 6–24 months are an appropriate time window. The similar rates of revictimisation observed in the studies with different follow‐up periods can be interpreted in multiple ways. It could be that the window of 6–24 months is the most important window for revictimisation. It could also be that we observed no effect of the duration of the follow‐up period because the studies with longer follow‐up periods differed from the studies with shorter follow‐up periods on other variables that influence the risk of revictimisation.

### 4.8 Meta‐Regression for IPV Revictimisation

We conducted an exploratory meta‐regression in the subgroup of studies investigating IPV revictimisation since, based on our observations during the coding‐procedure, this was the most prevalent type of revictimisation and studies in this group shared some important characteristics. These shared characteristics are the dynamic between perpetrator and victim (intimate partners) and the predominant use of (a version of) the same measurement instrument for revictimisation, the CTS‐2 (M. A. Straus et al. 1996). The results of this meta‐regression were largely in line with those of the overall group. However, we could not conduct an analysis on the effect of proportion of females in the sample, since all studies on IPV revictimisation used all‐female samples. Moreover, we found a negative effect of the duration of the follow‐up period on the rate of revictimisation. This may be due to the disproportional influence of a study with a rather long follow‐up period (96 months), that reported a lower rate of revictimisation (27.4%; Cascardi 2016) and one study with a very short follow‐up period (1 month) that reported a rather high rate of revictimisation (71.0%; Kiefer et al. 2024). We call for studies on IPV revictimisation that also use mixed‐gender samples since especially people with a minority gender identity (non‐binary/transgender) are at increased risk for (sexual) revictimisation (Blackburn et al. 2023).

### 4.9 Lessons Learnt

Longitudinal studies on revictimisation in the context of PTSD are very heterogeneous in terms of measurement methods, samples, and observed rates of revictimisation. A potential consequence of this heterogeneity is the possibility that we are contributing to a body of literature that contains multiple true effect sizes, which makes comparisons across studies very difficult, near impossible. Interestingly, other studies that focused on sexual revictimisation reported heterogeneity estimates that are comparable to the estimates in the current study (*I* ^*2*^ = 94.8 and *I* ^*2*^ = 97.1, respectively; Walker et al. 2019). Therefore, we can exclude our focus on more than one type of revictimisation incident as an alternative explanation for the observed heterogeneity. Rather, it seems that studies on any type of revictimisation lack consensus in what constitutes a (reliable/valid) study design or revictimisation. This point has been illustrated earlier in this discussion, where studies on sexual revictimisation had greatly differing definitions on what constitutes sexual revictimisation. Moreover, no measurement instrument measures revictimisation, rather studies repeatedly administer some type of victimization measure and count those who have been victimised more than once. While this could be a valid approach, it is impaired by the fact that almost all the studies we included used an *adapted* version or a subscale of a validated questionnaire. Therefore, the version of the questionnaire that they used was not validated anymore. This is not per se a shortcoming of an individual study, rather it highlights the need for more consensus of (a) what exactly constitutes revictimisation and (b) the need for the development of a questionnaire that assesses revictimisation.

### 4.10 Future Directions

We call for studies that aim to establish consensus on what constitutes revictimisation so the field can move forward with the development of a validated measurement instrument for revictimisation. It would be advisable to consult experts in the field to reach a consensus on what they regard as important dimensions that need to be assessed in the context of revictimisation. Based on their input and the input of experts by experience, a standardized measure for revictimisation could be developed and validated across different populations (e.g., student samples, clinical samples, people living in shelters). Moreover, we advise studies in the meantime to use validated instruments, and not adapted versions or only subscales of these instruments for the assessment of revictimisation. Future studies assessing PTSD symptoms should use one of the golden‐standard instruments as they have been validated and enable researchers to compare the severity of PTSD symptoms across studies (PCL‐5 for self‐report, CAPS‐5 for clinician‐assessment; Blevins et al. 2015; Weathers et al. 2018). Moreover, studies to date have largely focused on all‐female samples and we call for more gender diversity in samples. Additionally, given the high prevalence of revictimisation in those with psychiatric disorders, we call for more studies in clinical samples.

### 4.11 Implications

The current study has implications for both researchers in the field of revictimisation and clinicians working with groups vulnerable for revictimisation. For researchers, we have put forth recommendations that could enable the field to move forward with more consensus and thus enable future meta‐science to make clearer comparisons across studies. For clinicians, we have demonstrated that those who have been victimised before, either physically, sexually, by an intimate partner, or a combination thereof, are vulnerable to revictimisation. A possible way to address this issue would be to screen for prior victimization and provide psychoeducation about the risk of revictimisation. In short, we need to address revictimisation in psychotherapy and ensure that people know they are at increased risk. This conversation may open the discourse surrounding safety planning and/or the involvement of the social environment. Furthermore, it underlines the need for evidence‐based interventions aimed at preventing revictimisation (Table 5).

**TABLE 5:** Implications for practice, policy, and research.

Clinicians and researchers need to work together to develop a validated tool to measure revictimisation to enable comparability across studies and future meta‐science. Studies that claim to measure a phenomenon need to use validated measures for that phenomenon (e.g., golden standard assessments for PTSD/other psychopathology). We need more heterogeneity in samples (e.g., more males, non‐binary people, clinical populations) and less heterogeneity in measurement methods. Clinicians need to be aware that people with psychopathology (e.g., PTSD) are especially vulnerable to revictimisation. This should be addressed during treatment in an attempt to minimise revictimisation after treatment.

## Ethics Statement

Informed consent was obtained by participants during all studies that were included in the current article.

## Conflicts of Interest

The authors declare no conflicts of interest.

## Supporting information

Supporting Information S1

Supporting Information S2

Supporting Information S3

## Data Availability

All data and coding schemas can be obtained by requesting them from C.K.
