# Supplementary material for: Revictimisation Across Types of Interpersonal Violence: A Meta‐Regression Analysis of PTSD and Associated Factors
Source: Stress Health. 2025 Aug 6;41(4):e70079. doi: 10.1002/smi.70079 (PMC12326343; doi:10.1002/smi.70079)
Supplement: Supplementary file 1 — Supporting Information S1 [file SMI-41-e70079-s003.docx]

**Appendix 1**

| **Database searched** | **Platform** | **Years of coverage** | **Records** | **Records after duplicates removed** |
| --- | --- | --- | --- | --- |
| PubMed | PubMed | 1946 – Present | 419 | 419 |
| APA PsycInfo | EBSCO | 1968 - Present | 432 | 184 |
| PTSDpubs | Proquest | 1871 - Present | 404 | 255 |
| Web of Science | Clarivate | 1975 - Present | 746 | 342 |
| Scopus | Elsevier | 1970 - Present | 567 | 86 |
| **Total** | | | 2.568 | 1.286 |

Deduplication is performed using DedupEndnote (Lobbestael, G. (2023). DedupEndNote (Version 0.9.7e) [Computer software]. https://github.com/globbestael/DedupEndNote) and a manual check on title and authors by Medical Informationspecialist (CP).

|  | PubMed |  |
| --- | --- | --- |
| Search | Query | Results |
| #3 | Search: #1 AND #2 | 419 |
| #2 | Search: "Stress Disorders, Traumatic"[Mesh] OR "Adult Survivors of Child Abuse"[Mesh] OR "post-traumatic stress disorder*"[tiab] OR "posttraumatic stress disorder*"[tiab] OR PTSD[tiab] OR PTSS[tiab] OR "post traumatic neuros*"[tiab] OR "posttraumatic neuros*"[tiab] OR "psychological trauma*"[tiab] OR "psychotrauma*"[tiab] OR "complex trauma*"[tiab] OR "emotional trauma*"[tiab] OR "hyperarousal"[tiab] OR "avoidance"[tiab] OR "intrusion"[tiab] OR ("negative changes"[tiab] AND (mood[tiab] OR cognition[tiab])) OR (("disorder"[tiab] OR "disorders"[tiab]) AND ("post-traumatic"[tiab] OR "posttraumatic"[tiab])) OR "post-traumatic stress disorder"[tiab] OR "posttraumatic stress disorder"[tiab] OR "post-traumatic stress disorders"[tiab] OR "posttraumatic stress disorders"[tiab] OR "post-traumatic stress symptom*"[tiab] OR "posttraumatic stress symptom*"[tiab] OR "post-traumatic stress syndrome*"[tiab] OR "posttraumatic stress syndrome*"[tiab] OR "combat disorder*"[tiab] OR "shell shock*"[tiab] OR "combat neuros*"[tiab] OR "war neuros*"[tiab] OR (("adult survivor*"[tiab]) AND ("child abus*"[tiab] OR "childhood abus*"[tiab] OR "sexual abus*"[tiab] OR "child maltreatment*"[tiab] OR "child neglect*"[tiab] OR "Adverse childhood experien*"[tiab] OR "childhood adversit*"[tiab])) | 171,052 |
| #1 | Search: revictimization[tiab] OR "re-victimization"[tiab] OR revictimisation[tiab] OR "re-victimisation"[tiab] OR (repeated[tiab] AND (violen*[tiab] OR victimization[tiab] OR victimisation[tiab])) | 1,854 |

Key: [Mesh]= medical subject heading, [tiab] = title, abstract, author supplied keywords.

|  | **APA PsycInfo (EBSCO)** |  |  |
| --- | --- | --- | --- |
| **#** | **Query** | **Limiters/Expanders** | **Results** |
| **S4** | S1 AND S2 | Limiters – Academic journals | 432 |
| **S3** | S1 AND S2 | Search modes - Boolean/Phrase | 649 |
| **S2** | DE "Posttraumatic Stress" OR DE "Complex PTSD" OR DE "Posttraumatic Stress Disorder" OR DE "DESNOS" OR DE "Complex Trauma" OR DE "Acute Stress Disorder" OR DE "Emotional Trauma" OR DE "Traumatic Experiences" OR DE "Stress and Trauma Related Disorders" OR ((DE "Survivors") AND (DE "Child Abuse" OR DE "Child Neglect" OR DE "Emotional Abuse" OR DE "Physical Abuse" OR DE "Sexual Abuse" OR DE "Childhood Adversity")) OR TI("post-traumatic stress disorder*" OR "posttraumatic stress disorder*" OR PTSD OR PTSS OR "post traumatic neuros*" OR "posttraumatic neuros*" OR "psychological trauma*" OR "psychotrauma*" OR "complex trauma*" OR “emotional trauma*” OR "hyperarousal" OR "avoidance" OR "intrusion" OR ("negative changes" AND (mood OR cognition)) OR (("disorder" OR "disorders") AND ("post-traumatic" OR "posttraumatic")) OR "post-traumatic stress disorder" OR "posttraumatic stress disorder" OR "post-traumatic stress disorders" OR "posttraumatic stress disorders" OR "post-traumatic stress symptom*" OR "posttraumatic stress symptom*" OR "post-traumatic stress syndrome*" OR "posttraumatic stress syndrome*" OR "combat disorder*" OR "shell shock*" OR "combat neuros*" OR "war neuros*" OR (("adult survivor*") AND ("child abus*" OR "childhood abus*" OR "sexual abus*" OR "child maltreatment*" OR “child neglect*” OR “Adverse childhood experien*” OR “childhood adversit*”))) OR AB("post-traumatic stress disorder*" OR "posttraumatic stress disorder*" OR PTSD OR PTSS OR "post traumatic neuros*" OR "posttraumatic neuros*" OR "psychological trauma*" OR "psychotrauma*" OR "complex trauma*" OR “emotional trauma*” OR "hyperarousal" OR "avoidance" OR "intrusion" OR ("negative changes" AND (mood OR cognition)) OR (("disorder" OR "disorders") AND ("post-traumatic" OR "posttraumatic")) OR "post-traumatic stress disorder" OR "posttraumatic stress disorder" OR "post-traumatic stress disorders" OR "posttraumatic stress disorders" OR "post-traumatic stress symptom*" OR "posttraumatic stress symptom*" OR "post-traumatic stress syndrome*" OR "posttraumatic stress syndrome*" OR "combat disorder*" OR "shell shock*" OR "combat neuros*" OR "war neuros*" OR (("adult survivor*") AND ("child abus*" OR "childhood abus*" OR "sexual abus*" OR "child maltreatment*" OR “child neglect*” OR “Adverse childhood experien*” OR “childhood adversit*”))) OR KW("post-traumatic stress disorder*" OR "posttraumatic stress disorder*" OR PTSD OR PTSS OR "post traumatic neuros*" OR "posttraumatic neuros*" OR "psychological trauma*" OR "psychotrauma*" OR "complex trauma*" OR “emotional trauma*” OR "hyperarousal" OR "avoidance" OR "intrusion" OR ("negative changes" AND (mood OR cognition)) OR (("disorder" OR "disorders") AND ("post-traumatic" OR "posttraumatic")) OR "post-traumatic stress disorder" OR "posttraumatic stress disorder" OR "post-traumatic stress disorders" OR "posttraumatic stress disorders" OR "post-traumatic stress symptom*" OR "posttraumatic stress symptom*" OR "post-traumatic stress syndrome*" OR "posttraumatic stress syndrome*" OR "combat disorder*" OR "shell shock*" OR "combat neuros*" OR "war neuros*" OR (("adult survivor*") AND ("child abus*" OR "childhood abus*" OR "sexual abus*" OR "child maltreatment*" OR “child neglect*” OR “Adverse childhood experien*” OR “childhood adversit*”))) | Search modes - Boolean/Phrase | 140,634 |
| **S1** | TI(revictimization OR "re-victimization" OR revictimisation OR "re-victimisation" OR (repeated AND (violen* OR victimization OR victimisation))) OR AB(revictimization OR "re-victimization" OR revictimisation OR "re-victimisation" OR (repeated AND (violen* OR victimization OR victimisation))) OR KW(revictimization OR "re-victimization" OR revictimisation OR "re-victimisation" OR (repeated AND (violen* OR victimization OR victimisation))) | Search modes - Boolean/Phrase | 2,638 |

Key: DE= Descriptors,TI= title, AB= abstract and KW= Searches for keywords in the uncontrolled content description of the document

|  | **PTSDpubs (Proquest)** |  |
| --- | --- | --- |
| **#** | **Query** | **Results** |
| #3 | #2 AND filter Adults | 404 |
| #2 | #1 AND filter Scholarly journals | 714 |
| #1 | MAINSUBJECT.EXACT.EXPLODE("Revictimization") OR noft(revictimization OR "re-victimization" OR revictimisation OR "re-victimisation") OR noft((repeated AND (violen* OR victimization OR victimisation))) | 867 |

Key: MAINSUBJECT.EXACT.EXPLODE = Thesausus term; noft=Anywhere exept full text

|  | **Web of Science (Core collection) - Clarivate** |  |
| --- | --- | --- |
| **Nr.** | **Query** | Results |
| **#4** | #2 AND #1 and Article or Early Access or Proceeding Paper (Document Types) | 746 |
| #3 | #2 AND #1 | 821 |
| #2 | TS=("post-traumatic stress” OR PTSD OR PTSS OR "post traumatic neuros*" OR "posttraumatic neuros*" OR "psychological trauma*" OR "psychotrauma*" OR "complex trauma*" OR “emotional trauma*” OR "hyperarousal" OR "avoidance" OR "intrusion" OR ("negative changes" AND (mood OR cognition)) OR (("disorder" OR "disorders") AND ("post-traumatic" OR "posttraumatic")) OR "post-traumatic stress disorder" OR "posttraumatic stress disorder" OR "post-traumatic stress disorders" OR "posttraumatic stress disorders" OR "post-traumatic stress symptom*" OR "posttraumatic stress symptom*" OR "post-traumatic stress syndrome*" OR "posttraumatic stress syndrome*" OR "combat disorder*" OR "shell shock*" OR "combat neuros*" OR "war neuros*" OR (("adult survivor*") AND ("child abus*" OR "childhood abus*" OR "sexual abus*" OR "child maltreatment*" OR “child neglect*” OR “Adverse childhood experien*” OR “childhood adversit*”))) | 320,522 |
| #1 | TS=(revictimization OR "re-victimization" OR revictimisation OR "re-victimisation" OR (repeated AND (violen* OR victimization OR victimisation))) | 5,022 |

Key: TS = topic, which includes title, abstract, author keywords and Web of Science Keywords Plus.

|  | Scopus (Elsevier) |  |
| --- | --- | --- |
| History Count | Search Terms | Results |
| #4 | #3 AND ( LIMIT-TO ( DOCTYPE , "ar" ) OR LIMIT-TO ( DOCTYPE , "le" ) OR LIMIT-TO ( DOCTYPE , "no" ) OR LIMIT-TO ( DOCTYPE , "sh" ) ) ... | 567 |
| #3 | #1 AND #2 | 682 |
| #2 | TITLE-ABS-KEY ( "post-traumatic stress" OR ptsd OR ptss OR "post traumatic neuros*" OR "posttraumatic neuros*" OR "psychological trauma*" OR "psychotrauma*" OR "complex trauma*" OR "emotional trauma*" OR "hyperarousal" OR "avoidance" OR "intrusion" OR ( "negative changes" AND ( mood OR cognition ) ) OR ( ( "disorder" OR "disorders" ) AND ( "post-traumatic" OR "posttraumatic" ) ) OR "post-traumatic stress disorder" OR "posttraumatic stress disorder" OR "post-traumatic stress disorders" OR "posttraumatic stress disorders" OR "post-traumatic stress symptom*" OR "posttraumatic stress symptom*" OR "post-traumatic stress syndrome*" OR "posttraumatic stress syndrome*" OR "combat disorder*" OR "shell shock*" OR "combat neuros*" OR "war neuros*" OR ( ( "adult survivor*" ) AND ( "child abus*" OR "childhood abus*" OR "sexual abus*" OR "child maltreatment*" OR "child neglect*" OR "Adverse childhood experien*" OR "childhood adversit*" ) ) ) ... | 491,913 |
| #1 | TITLE-ABS-KEY ( revictimization OR "re-victimization" OR revictimisation OR "re-victimisation" OR ( repeated AND ( violen* OR victimization OR victimisation ) ) ) | 4,048 |

Key: TITLE, ABS and KEY searches in title, abstract, and author supplied keywords
