# Supplementary material for: Revictimisation Across Types of Interpersonal Violence: A Meta‐Regression Analysis of PTSD and Associated Factors
Source: Stress Health. 2025 Aug 6;41(4):e70079. doi: 10.1002/smi.70079 (PMC12326343; doi:10.1002/smi.70079)
Supplement: Supplementary file 3 — Supporting Information S3 [file SMI-41-e70079-s002.docx]

**Appendix 3**

*Overview of Risk of Bias Assessment for the N = 19 Included Studies*

| Authors, Year | Sampling Frame Bias | Responder Bias | Information Bias |
| --- | --- | --- | --- |
| Banyard et al., 2002 | high | high | high |
| Bell et al., 2008 | high | low | high |
| Cascardi, 2016 | high | unclear | high |
| Cole et al., 2008 | high | unclear | high |
| Dardis et al., 2018 | high | high | low |
| Dokkedahl et al., 2022 | low | high | high |
| Iverson et al., 2013 | high | unclear | high |
| Kiefer et al., 2024 | high | unclear | high |
| Krause et al., 2006 | high | unclear | high |
| Kuijpers et al., 2012 | high | high | high |
| Kunst et al., 2010 | high | high | high |
| Littleton et al., 2009 | high | unclear | high |
| Littleton et al., 2017 | high | unclear | high |
| Lowe et al., 2014 | low | high | high |
| Najdowski & Ullman, 2009 | high | high | low |
| Perez et al., 2012 | high | unclear | low |
| Perez & Johnson, 2008 | low | high | high |
| Scoglio et al., 2022 | high | high | high |
| Stockdale et al., 2014 | high | low | high |
